# Parent-Adolescent Conflict, Depressive Symptoms, and Non-Suicidal Self-Injury among Chinese Adolescents: The Moderating Effect of the COMT Gene rs4680 Polymorphism

**DOI:** 10.3390/ijerph191710567

**Published:** 2022-08-25

**Authors:** Yuting Deng, Meijin Li, Huahua Wang, Jingjing Li, Xu He, Chengfu Yu

**Affiliations:** 1 Department of Psychology, Research Center of Adolescent Psychology and Behavior, School of Education, Guangzhou University, Guangzhou 510006, China; 2 School of Psychology, South China Normal University, Guangzhou 510631, China

**Keywords:** parent-adolescent conflict, non-suicidal self-injury (NSSI), depressive symptoms, the COMT gene rs4680 polymorphism, adolescent

## Abstract

Existing research suggests that parent-adolescent conflict is associated with increased risk for adolescent non-suicidal self-injury (NSSI). However, adolescent NSSI reactions to parent-adolescent conflicts exhibit large individual differences. This study sought to explore whether depressive symptoms mediates the relationship between parent-adolescent conflict and adolescent NSSI, and whether this mediating process is moderated by the COMT gene rs4680 polymorphism. A total of 673 adolescents (364 males, 309 females) in the age range of 12 to 15 years (*Mean*_age_ = 12.81 years, *SD* = 0.48) completed questionnaires regarding parent-adolescent conflict, depressive symptoms, and NSSI. Genomic DNA was extracted from saliva and buccal cells from each participant. Bootstrapping techniques displayed statistically significant moderated mediation. The results showed that the positive association between parent-adolescent conflict and adolescent NSSI was in fact mediated by depressive symptoms. Moreover, this indirect link was moderated by the COMT gene rs4680 polymorphism. Specifically, the risk effect of parent-adolescent conflict on adolescent NSSI via depressive symptoms was stronger for adolescents with Val/Val genotype than for those with Met/Met or Val/Met genotype. These findings underscore the importance of examining the interaction between genes and the environment to understand how and when parent-adolescent conflict impacts adolescent NSSI.

## 1. Introduction

Non-suicidal self-injury (NSSI) refers to intentional destruction of body tissue (i.e., self-cutting, biting, and scratching skin) without the idea of suicide [1]. The prevalence of NSSI among adolescents in the past six months was 15% in China [2]. Furthermore, the rate of NSSI increased gradually among Chinese females after the age of 12 [3]. Adolescents with NSSI are more likely to develop depressive symptoms and suicidal ideation, and are at a risk of attempting suicide [4,5,6]. It is necessary to explore potential risk factors and mechanisms of NSSI.

Recently, the relationship between parent-adolescent conflict and NSSI has drawn increasing attention. According to the pragmatic hypothesis of the integrated theoretical model of NSSI, NSSI is an effective and simple means of immediately regulating distasteful emotional experiences and social situations [7]. NSSI is more likely to occur when individuals are experiencing interpersonal conflicts. It is reasonable to speculate that adolescents may adopt NSSI to release negative emotions when in conflict with their parents. Adolescents who reported using NSSI in the last six months, also described feeling alienated from parents [8]. Incidentally, Liang et al. found that adolescents with NSSI reported more conflicts with their parents [3]. These theories and empirical studies highlighted parent-adolescent conflict as an important risk predictor of adolescent NSSI.

### 1.1. The Mediating Effect of Depressive Symptoms

The affect-regulation model of self-injury proposes that self-injury is a maladaptive way of reacting to acute negative emotional arousal [9]. At the same time, negative emotional arousal such as depressive emotion often first emerges in adolescence [10]. Existing evidence supports this hypothesis by indicating a positive association between depressive symptoms and NSSI [11,12]. Moreover, Valencia-Agudo et al. retrieved 39 studies on NSSI indicated that depressive symptoms is observed to prospectively predict future NSSI [13]. Furthermore, preliminary research suggested that depressive symptoms played an important mediating role between family risk factors (e.g., parental rejection, harsh parenting) and adolescent NSSI [14,15,16]. A longitudinal research by Zhu et al., with a sample of 1987 Chinese adolescents, found that parental rejection predicted adolescents NSSI six months later through depressive symptoms [14]. Parent-adolescent conflict may be associated with NSSI via depressive symptoms and not via direct association. Parent-adolescent conflict is a specific form of interpersonal hostility, and this proximal factor can contribute to adolescents’ emotional problems (i.e., hopelessness and depression) [17], which in turn increases the risk of NSSI. Moreover, parent-adolescent conflict couldn’t provide a secure psychosocial resource (i.e., psychosocial support) protecting adolescents from depressive symptoms [18], and thus contributed to NSSI. The association between high parent-adolescent conflict and elevated depressive symptoms has been demonstrated by past studies [19,20,21]. Based on the theory and literature mentioned above, we propose the following hypothesis:

**Hypothesis** **1** **(H1).** *Depressive symptoms will mediate the link between parent-adolescent conflict and adolescent NSSI*.

### 1.2. The Moderating Effect of COMT Gene rs4680 Polymorphism

Although parent-adolescent conflict is considered as a significant risk factor for the development of NSSI, not all adolescents are equally influenced by parent-adolescent conflict. Based on the differential susceptibility model [22], adolescents with certain genotypes (i.e., Val/Val of rs4680) were not only more likely to be affected by negative environments and produce psychological and behavioral problems, but were also more likely to be affected by positive environments and showed more positive developmental results. One of the most frequently explored genes associated with dopamine is Catechol-O-Methyltransferase (COMT) gene (responsible for encoding the COMT enzyme). Compared to the Val/Val, Met allele of COMT rs4680 polymorphism has less enzymatic activity [23]. Furthermore, this enzyme may contribute to their effects on dopamine degradation [24] and the emotional function of the brain [25,26]. For instance, Vai et al. have pointed out that the positive functional connectivity between the amygdala and dorsolateral prefrontal cortex, which involves a high-order top-down regulation of affect, suggested an inefficient modulation in depressed bipolar patients who are homozygous for the Val allele [26]. Kotyuk et al. also found that rs4680 Met/Met genotype significantly associated with lower neuroticism level [27]. Previous studies have reported that rs4680 polymorphism interacts with the environment to influence mood dysregulation [28] and adolescents’ NSSI [15]. Sheikh and his colleagues showed the interaction effect between rs4680 and life stress on anxiety symptoms [28]. The result showed that under high levels of stress, carriers of the Val allele had significantly higher symptoms of anxiety compared to the Met allele carriers. Analogously, a previous study also found a significant alleviative effect of the Met allele on depression in the children with institutional care experience [29]. Liu et al., in a three way longitudinal study, emphasized that rs4680 moderated the relationship between harsh parenting and adolescents NSSI and the link between harsh parenting and depressive symptoms [15]. Particularly, adolescents with the Val/Val genotype showed more depressive symptoms and at a higher risk for NSSI when they experienced more harsh parenting, and appeared less depressed and at a lower risk for NSSI when experiencing lenient parenting. Based on this theory and existing empirical studies, we propose Hypothesis 2:

**Hypothesis** **2** **(H2).** *The rs4680 polymorphism will moderate the positive indirect link between parent-adolescent conflict and adolescent NSSI via depressive symptoms. This indirect association will be significant among adolescents with Val/Val genotype but much weaker among adolescents with Met/Met or Val/Met genotype.*

### 1.3. The Present Study

**Figure 1:**
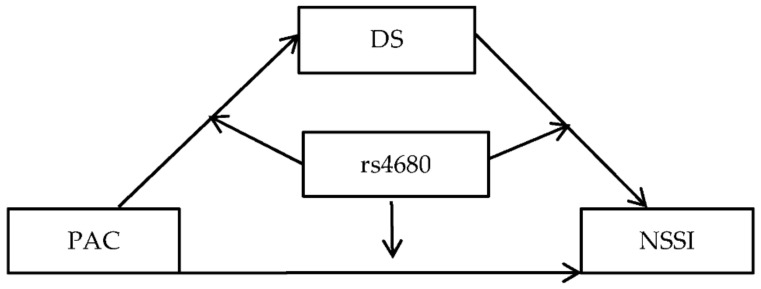
Hypothesized research model Note: DS = depressive symptoms, PAC = parent-adolescent conflict, NSSI = non-suicidal self-injury.

A moderated mediation model was designed to explore the role of depressive symptoms and COMT rs4680 in the relationship of parent-adolescent conflict and adolescent NSSI. Figure 1 illustrates the proposed research model.

## 2. Methods

### 2.1. Participants

In this study, cluster sampling method was used to choose subjects. 673 students in the age range of 12 to 15 years old were recruited as subjects from two middle schools in Guangdong province (*Mean*_age_ = 12.81 years, *SD* = 0.48 years). There were 364 adolescent male participants and 309 adolescent female participants.

### 2.2. Measures

#### 2.2.1. Parent-Adolescent Conflict

In this study, the conflict frequency subscale of the Parent-Child Conflict Questionnaire revised by Fang et al. [30] was used for assessment. In a total of eight items, teens were asked to report how often they had conflicts with their parents over school, housework, friends, and other issues in the past six months. A five-point Likert scale was used, with 1 for none and 5 for several times a day. Average scores were calculated for every single item. Higher scores indicated more frequent parent-child conflicts. The Cronbach’s *α* coefficient for this scale was 0.86.

#### 2.2.2. Depressive Symptoms

In this study, Center for Epidemiological Studies Depression Scale was used to measure in Chinese adolescents [31]. This scale has good reliability and construct validity in the measurement of depressive symptoms among non-clinical adolescents [32,33]. Adolescents were asked to report how often they were depressed in the past week. There were 20 items in total on a four-point Likert scale with scores ranging from 1 = “none” and 4 = “most of the time”. The average score of every single item was calculated. The higher the score, the severer the depressive symptoms. The Cronbach’s α coefficient for this scale was 0.84.

#### 2.2.3. NSSI

Adolescents were required to assess their frequency of participating in the six NSSI behaviors (i.e., self-directed cutting, engraving on the skin with a sharp object, scratching skin, pulling hair, bitting, rubbing the skin so hard as to bleed) in the past six months. These NSSI behaviors are relatively common among adolescents [1] and have been used to measure NSSI in previous studies[2,34]. All items were rated on a six-point Likert scale with 1 = “never” and 6 = “several times a week”. The average score of every single item was calculated. The higher the score, the more NSSI. The Cronbach’s *α* coefficient for this scale was 0.70.

### 2.3. The COMT Gene rs4680 Polymorphism

Genomic DNA was extracted from the saliva and buccal cells of each participant. Magnetic Bead Method Nucleic Acid Extraction Kit providing by a commercial company in China was used for DNA extraction. Single nucleotide polymorphism (SNP) genotyping was performed by the Wuhan Tianyi Huiyuan Biotechnology Co., Ltd., Wuhan, China. The detection rate of genotyping was 98.54%. Genotyping the COMT gene rs4680 polymorphism yielded three groups as follows: 35 were with Met/Met genotype, 276 were with Val/Met genotype, and 362 were with Val/Val genotype. The *χ*^2^ goodness of fit test showed that the genotype distribution of the COMT gene rs4680 polymorphism was consistent with Hardy-Weinberg equilibrium (*χ*^2^ = 3.65, *p* > 0.05).

### 2.4. Control Variables

Considering the significant impact of gender [35,36], age [37], and family financial difficulty [38] on adolescent depressive symptoms and NSSI, these were included as the control variables in this study. Family financial difficulty was measured with Chinese version of the Family Financial Difficulty Scale [39]. Participants were required to report how often their families had experienced financial stress during the last six months. A five-point Likert scale was used with 1 = “never” and 5 = “always”. The Cronbach’s *α* coefficient for this scale was 0.84.

### 2.5. Research Procedures and Statistical Analysis

Before the research, we obtained approval from the Ethics in Human Research Committee of the School of Education, Guangzhou University (date of approval: 15 January 2018). Second, with the consent of the parents and students, the experimenter distributed questionnaires to the students in their classes. Third, students were required to collect their saliva to saliva collector. The whole process of filling out the questionnaire and saliva collection took 45 min.

SPSS 20.0 was used for descriptive statistical analysis. We then used the Process Macro Model 4 for mediation analysis and Model 59 for moderated mediation model analysis [40]. Next, to reveal the moderating effect of depressive symptoms, the Johnson-Neyman(J-N) technique was used to examine the significant regions [41]. In comparison with the pick-a-point technique, the J-N technique offers more comprehensive details for showing how the effect of the parent-adolescent conflict on depressive symptoms depends on the value of rs4680 [42].

## 3. Results

### 3.1. Descriptive Statistics

The item mean score of family financial difficulty, parent-adolescent conflict, depressive symptoms and NSSI were 1.33 (*SD* = 0.57), 1.93 (*SD* = 0.82), 1.70 (*SD* = 0.43) and 1.07 (*SD* = 0.23), respectively. Table 1 provides the correlations between the research variables. As shown in Table 1, parent-adolescent conflict was positively associated with depressive symptoms and NSSI, and depressive symptoms was positively associated with NSSI. However, the relationship between the COMT gene rs4680 polymorphism, and depressive symptoms and NSSI was not significant.

### 3.2. The Mediating Effect of Depressive Symptoms

Mediation tests were conducted using the PROCESS macro for SPSS (model 4, with 1000 bootstrapped samples and bias-corrected 95% confidence intervals). This mediation model (Table 2) examed the link between parent-adolescent conflict (X), depressive symptoms (M), and NSSI(Y). After controlling for gender, age, and family financial difficulty, it was found that parent-adolescent conflict positively predicted depressive symptoms (*β* = 0.17, *SE* = 0.04, *t* = 4.38, *p* < 0.001, 95% CI [0.09, 0.24]), and depressive symptoms positively predicted NSSI (*β* = 0.27, *SE* = 0.04, *t* = 7.11, *p* < 0.001, 95% CI [0.20, 0.35]). The residual effect of parent-adolescent conflict on NSSI was significant (*β* = 0.08, *SE* = 0.04, *t* = 2.10, *p* < 0.05, 95% CI [0.01, 0.15]). The bias-corrected percentile bootstrap method showed that the mediating effect of depressive symptoms between parent-adolescent conflict and adolescent NSSI was significant (indirect effect = 0.0448, *SE* = 0.0146, 95% CI [0.0226, 0.0804]).

### 3.3. Testing for the Moderated Mediation Model

**Figure 2:**
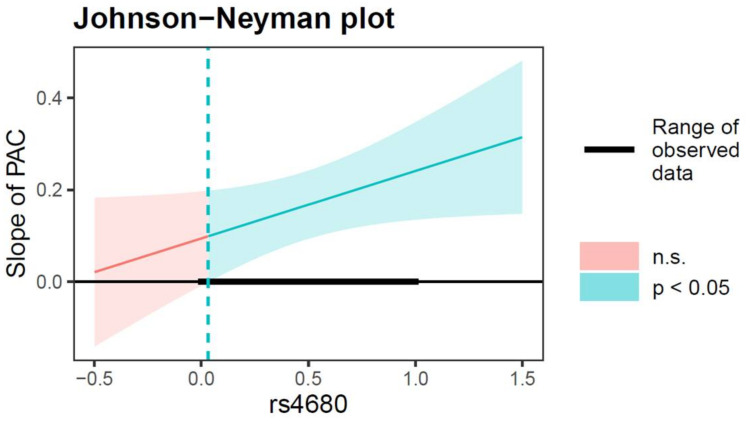
Interactive effect of PAC and rs4680 polymorphism on adolescent depressive symptoms. The green-red line indicates the simple slope of PAC contribute to depressive symptoms. Note: The green line represents the region of significance for the conditional association. Shaded area = confidence interval, PAC = parent-adolescent conflict, n.s. = not significant, the rs4680 value (1 = Val/Val, 0 = Val/Met, Met/Met).

**Figure 3:**
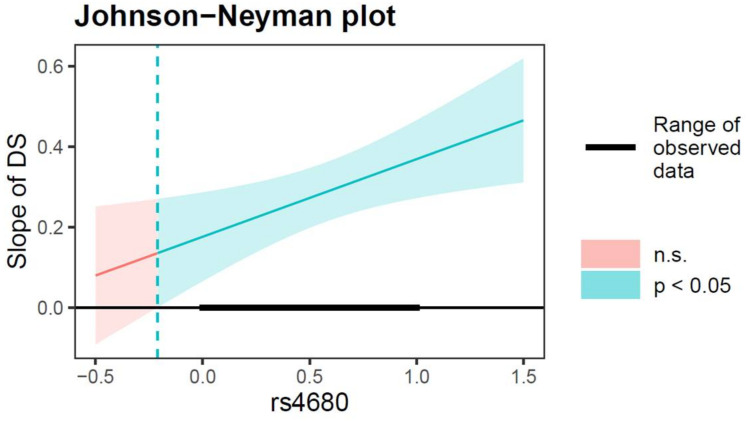
Interactive effect of DS and rs4680 polymorphism on adolescent NSSI. The green-red line indicates the simple slope of DS contribute to NSSI. Note: The green line represents the region of significance for the conditional association. Shaded area = confidence interval, DS = depressive symptoms, n.s. = not significant = CI [−5.47, −0.21], the rs4680 value (1 = Val/Val, 0 = Val/Met, Met/Met).

The moderated mediation model tests were conducted using the PROCESS macro for SPSS (model 59, with 1000 bootstrapped samples and bias-corrected 95% CI). The results are presented in Table 3. As shown in Equation 1, after controlling for covariates (gender, age, and family financial difficulty), the results showed that the interaction of parent-adolescent conflict and the COMT gene rs4680 polymorphism on depressive symptoms was significant (*β* = 0.15, *SE* = 0.07, *t* = 1.97, *p* < 0.05, 95% CI [0.0003, 0.29]). As shown in Figure 2, the positive association between parent-adolescent conflict and depressive symptoms was significant for rs4680 values greater than 0.03 for which the confidence bands do not contain zero. Thus, the adverse impact of parent-adolescent conflict on depressive symptoms was significant only in adolescents with the Val/Val genotype (rs4680 = 1). Moreover, as shown in Equation 2, the results showed that the main effects of depressive symptoms on NSSI were significant (*β* = 0.17, *SE* = 0.06, *t* = 3.05, *p* < 0.01, 95% CI [0.06, 0.28]), and the interaction effect between depressive symptoms and the COMT gene rs4680 polymorphism on NSSI was significant (*β* = 0.17, *SE* = 0.08, *t* = 2.30, *p* < 0.05, 95% CI [0.03, 0.32]). As shown in Figure 3, the positive relationship between depressive symptoms and NSSI was significant when the rs4680 value is outside the interval [−5.47, −0.21]. Therefore, for Val/Val genotype adolescents (rs4680 = 1), higher depressive symptoms was significantly and positively associated with higher NSSI. Furthermore, for Met/Met and Val/Met genotype adolescents (rs4680 = 0), the effect of depressive symptoms on NSSI was weaker.

We further tested whether the indirect effect between parent-adolescent conflict and adolescent NSSI via depressive symptoms was conditioned by the COMT gene rs4680 polymorphism. The bias-corrected percentile bootstrap results indicated that the indirect effect was stronger for adolescents with Val/Val genotype (conditional indirect effect = 0.08, *SE* = 0.03, 95% CI [0.03, 0.17]) than for those with Met/Met or Val/Met genotype (conditional indirect effect = 0.02, *SE* = 0.01, 95% CI [0.003, 0.04]). Therefore, the mediation pathway “parent-adolescent conflict → depressive symptoms → NSSI” was moderated by the rs4680 polymorphism, which formed a moderated mediation model.

## 4. Discussion

The current study explored the moderated mediation mechanism through which parent-adolescent conflict impacts adolescent NSSI. Consistent with Hypothesis 1, this study found that parent-adolescent conflict was associated with depressive symptoms, which in turn triggered adolescent NSSI. This finding suggests that depressive symptoms may be an important expository mechanism for the relationship between parent-adolescent conflict and NSSI. This result is congruent with the experiential avoidance model [43] which postulates that conflict with parents elicits an emotional response (i.e., depressive symptoms) in order to escape to loathsome emotional experiences, which in turn prompted their NSSI. This result is consistent with findings from previous studies that a negative family environment is an important factor for adolescent NSSI via the mediating effect of depressive symptoms [14,16,44]. Apart from the overall mediation results, each and every individual link in the mediation model of depressive symptoms is noteworthy. First, for the path from parent-adolescent conflict to depressive symptoms, individuals with high level of parent-adolescent conflict have higher level of depressive symptoms. This result highlights the important influence of parent-adolescent relationship in adolescent depressive symptoms. As a common hostile stimulus, parent-adolescent conflict may lead to the personal emotional deregulation of adolescents [45], and then, trigger more depressive symptoms. Second, we also found that adolescents with a high level of depressive symptoms are more likely to engage in NSSI, which aligns with previous research [46]. Adolescents with depressive symptoms increased rumination and decreased acceptance and tolerance of negative affect and in turn experience increased need to engage in NSSI [47].

Moreover, consistent with Hypothesis 2, the findings showed that COMT rs4680 moderated the relationship between parent-adolescent conflict and adolescent depressive symptoms. Particularly, among adolescents with Val/Val genotype but not Met allele, parent-adolescent conflict allows a significantly positive prediction of depressive symptoms. This result is consistent with previous research that adolescents with Val/Val homozygotes may be more susceptible to negative family environments, which, in turn, may trigger depressive symptoms [15,29]. In a high level of parent-adolescent conflict, negative interactions with parents can affect subcortical limbic regions associated with emotional reactivity [48] and thus develop depressive symptoms. Furthermore, the results indicated that individuals with Val/Val genotype had reduced white matter in some brain areas (i.e., bilateral middle temporal gyrus, right frontal gyrus, and right cingulum bundle area), which suggests cortico-limbic network dysfunction of depression, compared with Met allele carriers [49]. In other words, Val/Val genotype, but not Met allele, intensifies the relationship between parent-adolescent conflict and adolescents depressive symptoms.

The findings also showed that rs4680 polymorphism significantly moderated the relationship between depressive symptoms and adolescent NSSI. Adolescents with Val/Val genotype but not Met allele, suffered higher levels of depressive symptoms, and were more vulnerable to NSSI. A previous study has found that Val is a predominant factor that determines low levels of dopamine in the prefrontal cortex [50], which is an important brain area of executive function [51]. Furthermore, a meta-analysis showed that healthy individuals with Val/Val homozygotes performed worse than those with Met allele in cognitive flexibility tasks [52]. Therefore, with a high level of depressive symptoms, adolescents with the Val/Val genotype exhibit lower cognitive flexibility, thus reinforcing the association between depressive symptoms and NSSI. In addition, findings showed that in the context of low levels of depressive symptoms, adolescents with the Val/Val genotype seem to have lower levels of NSSI than their counterparts with the Met allele. The current results provide support for the differential susceptibility model which suggests that some adolescents with susceptibility factors (i.e., Val/Val genotype) are more sensitive to the environment, that is, showing better outcomes in positive environment and worse outcomes in negative environment [22]. This result is in line with previous studies that give evidence for the adaptive performance of adolescents with Val/Val genotype in a positive context [53].

This study has several limitations that must be considered when interpreting the findings. First, because the study was cross-sectional, we cannot conclude that parent-adolescent conflict contributes to the development of adolescent NSSI. Therefore, a longitudinal study design can strengthen the causal examination of the concerned variables. Second, the variables in this study were self-reported by adolescents, which will have a recall bias. Future studies could measure variables from adolescents and their parents. Third, our results didn’t exclude the role of psychiatric comorbidities such as borderline personality disorder pathology [54] and anxiety [3] and other negative environment (i.e., bullying at school[55]), which have been found positive association with NSSI. Fourth, the scores of depressive symptoms in the study were low and difficult to interpret in light of the correlations. Finally, the candidate gene/interaction studies are so hard to replication and risk for false positives [56]. Future studies could adopt genomewide association study [57].

Despite having several limitations, the current study also has some valuable practical implications. First, It was found that parent-adolescent conflict is a risk factor for adolescent NSSI. Adaptive communication strategy (i.e., fully respect) might reduce the frequency of parent-adolescent conflict. Second, this study confirmed that parent-adolescent conflict could enhance the level of NSSI by increasing adolescent depressive symptoms, which provides worthy implication for future intervention. Acceptance without judgment, dimension of dialectical behaviour therapy, has been identified a useful skill for NSSI [58]. Finally, the moderated mediation model in this study demonstrated that integrated programs that think over both genetic and environmental factors contemporaneously are needed to prevent adolescent NSSI.

## 5. Conclusions

Limitations notwithstanding, in a sample of Chinese adolescents, our study demonstrated that the depressive symptoms significantly mediated the relationship between parent-adolescent conflict and adolescent NSSI. Furthermore, COMT rs4680 moderated the first and second half of the mediating effect of “parent-adolescent conflict—depressive symptoms—adolescent NSSI”. Specifically, the risk effect of parent-adolescent conflict on adolescent NSSI via depressive symptoms was stronger for adolescents with Val/Val genotype than for those with Met alleles.

## Figures and Tables

**Table 1:** Correlations between research variables.

	Gender	Age	FFD	PAC	DS	NSSI	rs4680
Gender	1.00						
Age	0.06	1.00					
FFD	0.03	0.03	1.00				
PAC	0.11 **	0.05	0.13 **	1.00			
DS	−0.05	0.04	0.21 ***	0.18 ***	1.00		
NSSI	−0.06	−0.01	0.13 **	0.13 ***	0.30 ***	1.00	
rs4680	−0.02	−0.03	−0.02	−0.04	−0.02	0.06	1.00

Note: ** *p* < 0.01, *** *p* < 0.001. Gender was dummy coded (1 = male, 0 = female), the COMT gene rs4680 was dummy coded (1 = Val/Val, 0 = Val/Met, Met/Met), FFD = family financial difficulty, PAC = parent-adolescent conflict, DS = depressive symptoms, NSSI = non-suicidal self-injury.

**Table 2:** Testing for the mediation effect of depressive symptoms.

	Equation 1 (DS)				Equation 2 (NSSI)			
	*β*	*SE*	*t*	95% CI	*β*	*SE*	*t*	95% CI
Covariates:								
Gender	−0.16	0.08	−2.11 *	[−0.31, −0.01]	−0.10	0.07	−1.38	[−0.25, 0.04]
Age	0.03	0.04	0.89	[−0.04, 0.11]	−0.02	0.04	−0.64	[−0.10, 0.05]
FFD	0.19	0.04	5.16 ***	[0.12, 0.27]	0.06	0.04	1.61	[−0.01, 0.13]
Study variables:								
PAC	0.17	0.04	4.38 ***	[0.09, 0.24]	0.08	0.04	2.10 *	[0.01, 0.15]
DS					0.27	0.04	7.11 ***	[0.20, 0.35]
*R* ^2^	0.08				0.10			
*F*	13.88 ***				15.26 ***			

Note: Values are standardized coefficients. * *p* < 0.05, *** *p* < 0.001. Gender was dummy coded (1 = male, 0 = female). FFD = family financial difficulty, PAC = parent-adolescent conflict, DS = depressive symptoms, NSSI = non-suicidal self-injury.

**Table 3:** Testing for the moderating role of rs4680 polymorphism on the indirect relationship between parent-adolescent conflict and adolescent NSSI via depressive symptoms.

	DS				NSSI			
	*β*	*SE*	*t*	95% CI	*β*	*SE*	*t*	95% CI
Covariates:								
Gender	−0.16	0.08	−2.11 *	[−0.31, −0.01]	−0.09	0.07	−1.23	[−0.24, 0.05]
Age	0.03	0.04	0.80	[−0.04, 0.10]	−0.03	0.04	−0.74	[−0.10, 0.05]
FFD	0.19	0.04	5.07 ***	[0.12, 0.26]	0.07	0.04	1.75	[−0.01, 0.14]
Study variables:								
PAC	0.09	0.05	1.37	[−0.01, 0.20]	0.05	0.05	0.93	[−0.05, 0.15]
rs4680	−0.01	0.07	−0.14	[−0.16, 0.14]	0.12	0.07	1.69	[−0.02, 0.27]
PAC × rs4680	0.15	0.07	1.97 *	[0.00, 0.29]	0.06	0.07	0.79	[−0.09, 0.21]
DS					0.17	0.06	3.05 **	[0.06, 0.28]
DS × rs4680					0.17	0.08	2.30 *	[0.03, 0.32]
*R* ^2^	0.08				0.12			
*F*	9.93 ***				10.85 ***			

Note: Values are standardized coefficients. * *p* < 0.05, ** *p* < 0.01, *** *p* < 0.001. Gender was dummy coded (1 = male, 0 = female), the COMT gene rs4680 was dummy coded (1 = Val/Val, 0 = Val/Met, Met/Met), FFD = family financial difficulty, PAC = parent-adolescent conflict, DS = depressive symptoms, NSSI = non-suicidal self-injury.
